# Pneumococcal Choline-Binding Proteins Involved in Virulence as Vaccine Candidates

**DOI:** 10.3390/vaccines9020181

**Published:** 2021-02-20

**Authors:** Julio Sempere, Mirella Llamosí, Idoia del Río Menéndez, Beatriz López Ruiz, Mirian Domenech, Fernando González-Camacho

**Affiliations:** 1 Spanish Pneumococcal Reference Laboratory, National Centre for Microbiology, Instituto de Salud Carlos III, 28220 Majadahonda, Spain; jsempere@isciii.es (J.S.); mirella.llamosi@isciii.es (M.L.); idoia.mnendz@gmail.com (I.d.R.M.); b.lopezruiz@isciii.es (B.L.R.); 2 Centro de Investigación Biomédica en Red de Enfermedades Respiratorias (CIBERES), 28029 Madrid, Spain

**Keywords:** *Streptococcus pneumoniae*, choline-binding proteins, pneumococcal vaccines, protein vaccines

## Abstract

*Streptococcus pneumoniae* is a pathogen responsible for millions of deaths worldwide. Currently, the available vaccines for the prevention of *S. pneumoniae* infections are the 23-valent pneumococcal polysaccharide-based vaccine (PPV-23) and the pneumococcal conjugate vaccines (PCV10 and PCV13). These vaccines only cover some pneumococcal serotypes (up to 100 different serotypes have been identified) and are unable to protect against non-vaccine serotypes and non-encapsulated pneumococci. The emergence of antibiotic-resistant non-vaccine serotypes after these vaccines is an increasing threat. Therefore, there is an urgent need to develop new pneumococcal vaccines which could cover a wide range of serotypes. One of the vaccines most characterized as a prophylactic alternative to current PPV-23 or PCVs is a vaccine based on pneumococcal protein antigens. The choline-binding proteins (CBP) are found in all pneumococcal strains, giving them the characteristic to be potential vaccine candidates as they may protect against different serotypes. In this review, we have focused the attention on different CBPs as vaccine candidates because they are involved in the pathogenesis process, confirming their immunogenicity and protection against pneumococcal infection. The review summarizes the major contribution of these proteins to virulence and reinforces the fact that antibodies elicited against many of them may block or interfere with their role in the infection process.

## 1. Introduction

*Streptococcus pneumoniae*, also called the pneumococcus, is a human pathogen that colonizes the nasopharynx of healthy people, mainly children (20–40% in children younger than 2 years) and occasionally adults (5–10%) [1]. Pneumococcus establishes a commensal relationship in the host’s nasopharynx and this is usually asymptomatic [2]. In certain circumstances, the equilibrium with the host is broken, being able to invade other niches causing different pathologies [3]. *S. pneumoniae* is responsible from more than 800,000 annual deaths in children younger than 5 years every year worldwide, especially in developing countries [4].

Nasopharyngeal colonization is a prerequisite for the establishment of the invasive pneumococcal disease (IPD) process, although it is also necessary for developing non-bacteremic pneumonia, otitis media and sinusitis [5]. The primary mechanism of transmission is via airborne droplets. In the first stage, the bacteria have to adhere to the mucosal epithelium of the host and colonize the nasopharynx. Once colonized, it can remain here or, by direct extension from the nasopharynx, reach the nasal sinuses, the Eustachian tube, the trachea or the bronchi where the bacteria will have to defend themselves from the natural clearance mechanisms by the host. Once this barrier is surpassed, it could cause acute otitis media (AOM), sinusitis, non-bacteremic pneumonia or empyema. More serious clinical manifestations can also occur when the bacteria have reached the blood or cerebrospinal fluid producing systemic infection (preceded or not by pneumonia) and meningitis. Other less common invasive diseases are myocardial inflammation, acute cardiac events, pericarditis and acute arthritis [6,7,8]. The IPD process is potentially fatal and in the case of meningitis is frequently associated with a high rate of serious sequelae in patients who survive the infectious process [9,10].

## 2. *Streptococcus pneumoniae* Pathogenesis

Pathogenicity is determined by the interactions established between the pneumococcus and the host. These interactions mainly involve most external components of the bacteria, such as the polysaccharide capsule and the components of the cell wall, although other internal components may also intervene in the pathogenesis that are released to the outside as toxic substances—pneumolysin (Ply) being one of the most important [2,3].

The capsule polysaccharide (CPS) is considered the main pneumococcal virulence factor; its primary role in virulence is to shield the cell wall from host antibodies and the complement, the CPS protects the bacteria against opsonophagocytosis [11,12,13]. The CPS is responsible for stimulating the production of specific antibodies for each serotype, and 100 different serotypes of *S. pneumoniae* [14] have been identified so far, although 20 of them are responsible for 96% and 89% of IPD cases in children under 2 years old and adults ≥65 years old, respectively [15]. Thus, the interactions between the capsule and host immunity likely vary with capsule type.

There are also non-encapsulated *S. pneumoniae* (NP*Sp*) strains, which are emerging due to the selective pressure that conjugate vaccines are producing on the vaccine serotypes that favor a replacement in the nasopharyngeal carrier state [16,17,18]. Moreover, NP*Sp* is underestimated because the typing clinical tests for characterization are based on the capsule. NP*Sp* strains are responsible for non-invasive diseases such as AOM [19], non-bacteremic pneumonia [20] and conjunctivitis [21,22,23] and rarely produce IPD [24,25]. On the other hand, it is common for many of these strains to show resistance to antibiotics [18,26,27], and they can participate in the global pathogenesis as a reservoir of resistance genes that can be transferred to capsulated strains [28]. Acquisition and expression of NE*Sp* virulence genes instead of capsule synthesis could provide vaccine-targeted pneumococci—an easily attainable alternative strategy for pathogenesis. A subset of group II NE*Sp* designated as null capsule clade 1 (NCC1) encodes the gene for pneumococcal surface protein K (*pspK*) in their cps locus [28]. It has been demonstrated that PspK aids in NCC1 nasopharyngeal colonization, which is a prerequisite to pneumococcal disease. PspK also increases adherence to lung epithelia and persistence within the middle ear [29,30]. The *pspK* acquisition could be a potential mechanism to evade PCV [31].

Furthermore, the pneumococcal cell wall is composed of teichoic acids, lipoteichoic acids and peptidoglycan capable of inducing an inflammatory response, as well as the activation of the alternative complement pathway [32,33]. Anchored to the cell wall, exposed to the bacterial surface, there are proteins that participate in the pathogenicity of the bacteria and are considered virulence factors due to the host-pathogen interaction. These surface proteins from *S. pneumoniae* have been grouped into four different protein families: CBPs (16 proteins), lipoproteins (50 proteins), proteins with the LPXTG sequence (18 proteins) and the non-classical surface protein family (NCSPs) (6 proteins) [34].

Other elements that contribute to the pneumococcal pathogenesis can come from the cell cytoplasm, producing toxic substances such as Ply, a cytolytic toxin, with hemolytic capacity [35,36].

## 3. Complement-Mediated Immunity

The complement system is an important defense mechanism of the innate immunity that respond quickly and non-specifically against microorganisms. More than 30 different proteins (and proteolytic fragments) from serum participate in the complement system; they intervene through a regulated process. Activation of the defense system plays a fundamental role in the fight against pneumococcus, leading to a cascade of processes that ends with a series of protein elements adhered to the surface of the bacteria, causing their opsonization and favoring subsequent phagocytosis (opsonophagocytosis). In addition, the complement system promotes an inflammatory response to infection [37].

The complement cascade can be activated by three different pathways: the classical pathway (CP) that is initiated by C1q, when binding to antibodies recognizing the bacterial surfaces (antigen-antibody complexes) or by direct binding to the bacterial surface. This activation mechanism is vital in innate immunity against capsulated bacteria such as the pneumococcus [38,39]. The second pathway is the lectin pathway, in which lectins recognize certain carbohydrates on the bacterial surface, activating this cascade in the absence of antibodies, making it important for the recognition of pneumococcus [40]. Finally, the alternative pathway (AP) is activated on the surface of microorganisms and does not require the binding of antibodies, and it is important against pneumococcus because it amplifies the activation of C3, which is the key complement component where all the three pathways converge [37,39].

The activity of the complement system is downregulated to avoid damage in host cells while efficient activation is allowed in bacteria. There are a variety of proteins to regulate the location and activity of complement [37,41]. Factor H (FH) is the downregulator of the AP that participates in the inactivation of C3b when it binds to the surfaces of the host’s cells, and this is a mechanism that this bacterium uses to evade the immune system [42]. Another negative regulator is the C4b-binding protein (C4BP) that participates in the inhibition of the classical and lectins pathways. This protein inhibits the formation of the C3 convertase. The complement regulatory activity of C4BP is also exploited by some pathogens which bind C4BP to their surfaces to evade the activity of the complement system [43].

The main result of the complement-mediated response to pneumococcus is the increase in opsonization and phagocytosis by macrophages and neutrophils, which can control the bacterial load and prevent its expansion. This complement-mediated phagocytosis is an essential defense mechanism in response to pneumococcal infection, controlling the spread of the bacteria in the IPD process [44,45].

The selective pressure of the complement system on the pneumococcus has led this microorganism to develop interference systems to avoid complement-mediated opsonophagocytosis. The main component of the bacteria that interferes with complement elements is the polysaccharide capsule, which acts by hindering and reducing the deposition of complement molecules and preventing phagocytosis [46,47].

In the case of *S. pneumoniae*, several bacterial proteins are involved in evasion of different steps of the complement system to avoid its role in host defense. Among these proteins, CBPs are a family of pneumococcal virulence factors with several members acting at different levels of the complement system in order to avoid and/or reduce its activation [42]. These defense mechanisms of the bacterium to avoid the complement system are crucial both for the persistence as a carrier state in the host and for the development of the infection. Targeting these proteins using vaccines that can block or interfere in the functionality of these proteins may become a critical key from the prophylactic perspective in the control of pneumococcal virulence and pathogenesis.

## 4. Current Commercialized Vaccines for *Streptococcus pneumoniae*

Currently, there are two kind of pneumococcal vaccines that are being used to prevent infection by this pathogen and both vaccines target the same virulence factor, which is CPS. The first vaccine commercialized was the 23-valent pneumococcal polysaccharide vaccine (PPV-23), and the second group of vaccines based on CPS are PCVs in which the CPS is conjugated to a carrier protein. PPV-23 is poorly immunogenic in children younger than two years. Moreover, PPV-23 neither develops immunological memory nor eliminates the carrier state; moreover, this vaccine might be effective in preventing the invasive forms of pneumococcal diseases, but protection against pneumonia is limited [48].

PCVs provide a T-Cell-dependent response with high immunogenicity and an efficient immune memory, therefore being recommended for the pediatric population under 2 years old. Among these sorts of vaccines, the first PCV commercialized was the 7-valent PCV (PCV7), replaced later by the 10-valent PCV (PCV10) and the 13-valent PCV (PCV13), which are currently used worldwide. Recent studies have demonstrated that PCV13 is effective not only against IPD but also against pneumonia and has effect on nasopharyngeal carriage providing herd immunity to the elderly population [49,50]. Reduction in the carrier state caused by serotypes included in PCVs has induced the phenomenon of herd protection which is a great achievement of these vaccines but also has contributed to serotype replacement; the main limitation of these sort of vaccines based on CPS [51,52,53].

The vaccination schedule and recommendations are based on age and risk factors [54]. PCVs are currently recommended for incorporation in the childhood vaccination program despite the limitations of covered serotypes and high cost of these vaccines. In people with certain underlying diseases, sequential vaccination with the two available vaccines (PPV-23 plus PCV) is recommended [55,56] and PPV-23 is recommended in the adult population over 60 years.

As previously mentioned, the main disadvantage of these vaccines is that they do not prevent disease caused by serotypes that are not included in current formulations or those caused by non-capsulated strains. Serotype replacement can become a serious problem for the control of the disease [15,53,57,58]. To solve these problems by current vaccines based in CPS as the target antigen, it is necessary to develop new vaccines that can provide immunological protection regardless of the serotype and provide universal protection against pneumococcus [59,60].

## 5. Current Strategies in the Development of Pneumococcal Proteins as Vaccine Candidates

There are different approaches to circumvent the limitations and problems described above in the characterization and development of alternative prophylactic vaccines to those based exclusively on CPS. The use of whole-cell pneumococcus, either based on live attenuated cells [61], inactivated whole cells, crude extracts [62,63,64] or in multi-antigenic acellular systems [65] are possibilities under research. A different approach, based on current strategies, would be to conjugate the polysaccharides to a pneumococcal protein carrier [66,67,68] to improve the immune response against the pathogen or, alternatively, to incorporate other elements into the formulation that increase this response [69,70].

One of the vaccines most characterized as a prophylactic alternative to current PPV23 or PCVs is the development of a vaccine based on pneumococcal protein antigens. In the case of *Neisseria meningitidis* serogroup B, there are actually two vaccines based on meningococcal proteins that are showing high efficacy rates against meningococcal disease caused by clinical isolates of serogroup B [71,72,73].

In the case of *S. pneumoniae*, several groups have characterized different proteins as vaccine candidates. Antibodies generated against these proteins may induce the cellular immune response through the opsonophagocytosis process, but they also may interfere with the function of the bacterial protein to which it binds. As a consequence, human antibodies against pneumococcal proteins, for example, could reduce pneumococcal adherence to epithelial cells and prevent nasopharyngeal colonization, or even reduce the interaction with lung epithelium, therefore avoiding, the development of pneumonia and systemic dissemination.

Pneumococcal strains can present different phenotypic characteristics depending on the physiological state and the environment in which they are found. For example, Ply, a well-known vaccine candidate, has greater relevance in the lung than in the process of nasopharyngeal colonization [74]. One characteristic that can increase the immunogenicity of the protein as antigen candidate is the location of the protein on the bacterial envelope in order to increase the interaction with essential components of the host immune response. Surface expose proteins might have increased immunogenic potential, inducing humoral and cellular responses that can block or interfere with the functionality of the protein as virulence factor.

Regarding nasopharyngeal colonization and the importance of NE*Sp* in this niche, Jang and colleagues demonstrated that R3 domain of PspK may be an effective vaccine candidate for both NE*Sp* and encapsulated *S. pneumoniae*. The immunization with R3 or UD: R3 of PspK elicited effective humoral and mucosal immune responses against nasopharyngeal colonization and the subsequent development of AOM and pulmonary infection in mice [75].

Proteins used in vaccines against pneumococcal disease must generate both humoral and cellular immunity. In order to be a candidate for a vaccine, a protein must contain well conserved antigenic regions avoiding sequence variability between the different serotypes. Most of the approaches focus on the use of purified, recombinant or fusion proteins [76]. These vaccines contain pneumococcal protein elements in their composition that act as universal antigens against all serotypes and are capable of inducing an immune response by recognizing both T-cell and B-cell receptors. The strategies are diverse: vaccines based on full-length protein [77] or the use of a single recombinant protein, for example, PhtD [78].

Some of the possible disadvantages of the use of a single protein is the low immune response and that the bacteria could evade its recognition with small changes in the structure. As an alternative, several recombinant proteins are used in the same formulation, achieving a complementary effect on the immune response [74].

To obtain optimal protection, the alternative could be a vaccine based on a chimeric construction, with multiple antigens that confer a greater degree of protection against the carrier state, pneumonia, bacteremia and meningitis.

In some of the candidate vaccine proteins, it is observed that the amino acid sequence between serotypes and their level of expression may vary, this variability means that the immune response is not equally efficient in all cases. To overcome this problem, vaccines that are based on the use of conserved epitopes are designed based only on highly conserved peptides [79] with antigenic capacity. The variability of virulence factors has been studied at the level genes comparative between strains [79]. For vaccine development, the proteins’ variability must be studied in order to choose the best epitopes to be included in the future vaccine.

There are various methods used to identify these antigenic regions of interest, which are not very variable and can protect against different manifestations of pneumococcal disease. It can be a genomic approach using the fingerprinting technique [80], or searching libraries of complete genomes [81], bioinformatics [82] or reverse vaccinology [83,84].

## 6. Choline-Binding Proteins as Vaccine Candidates

The CBPs are found in all pneumococcal strains [85], giving them the characteristic to be potential vaccine candidates as they may protect against different serotypes [77]. CBPs are the sum of two different modules: a functional module (FM) and a conserved choline-binding module (CBM); the last allows the non-covalent binding to phosphoryl choline (P-Cho) residues of teichoic and lipoteichoic acids of the pneumococcal cell wall [86]. With the exception of the murein hydrolases LytB and LytC, the CBM is located at the C-terminus, which in the case of PspA and PspC is preceded by a proline-rich linker [87]. The number of CBPs in *S. pneumoniae* depends on the strain but it is usually between 13 and 16 (Table 1) [85], and some of these proteins can be highly variable [88,89,90].

The functional relevance of CBPs in humans as possible immunogenic proteins was described evaluating patients recovering from IPD who elicited a specific immune response against some of the CBPs described above [80].

Proteins of the CBP family can be found in other commensal bacterial species with P-Cho residues in their cell wall such as *Streptococcus mitis*, *Streptococcus pseudopneumoniae* and *Streptococcus oralis* [115,116,117]. Vaccines based on these proteins can also target these organisms. It has been described that cell wall lytic CBPs are highly conserved among *S. pneumoniae* and *S. mitis*, while others CBPs, such as PspA, CbpA and PcpA, are species specific and show variation between pneumococcal strains [118]. Indeed, the design of vaccines based on epitopes should be taken into account that they could be conserved in the commensal microbiota and could alter the balance of these communities.

Furthermore, a worrisome aspect of protein vaccines that could alter the colonization by *S. pneumoniae* and other commensal bacterial species is the emergence of colonization by invasive pathogens such as *Staphylococcus aureus*. When you eliminate a population of bacteria from its preferred niche, this void could be filled by pathogens that could cause similar or worse diseases [119]. For instance, since the introduction of PCVs, some studies reported a negative association between PCV serotypes and *S. aureus* carriage [120,121,122], raising concerns about vaccines that target colonization and could prepare the niche for other pathogens to colonize. However, there are studies that showed that the vaccination with the conjugate vaccine did not yield a change on *S. aureus* nasopharyngeal carriage [123,124]. Some of the CBPs vaccine candidates affect the carrier state, which is an advantage, as vaccines with sterilizing immunity would eliminate the pathogen’s niche and eradicate most of the burden of disease; it is also a disadvantage, as these changes in the colonization could prepare the niche for even worse pathogens, at the same time.

### 6.1. CBPs with A Role in Immune Evasion as Vaccine Candidates

#### 6.1.1. LytA

The main cell wall hydrolase (CWH), LytA (*N*-acetylmuramoyl-L-alanine amidase), is the major autolytic enzyme of the bacterium, being involved in the lysis of the cell wall, therefore releasing the cytotoxin Ply and inflammatory mediators (teichoic acids and peptidoglycan fragments) [125]. Although two families of *lytA* alleles have been described, nucleotide polymorphism does not affect the primary structure of the protein, in which perfect amino acid sequence conservation was observed [126]. A direct role in host immune evasion was demonstrated for LytA, confirming that LytA avoids the recognition by C3b using a Ply-independent mechanism. In that study, using single and double mutants of LytA and Ply showed that LytA inhibited the activation of both the alternative and the classical pathway of the complement system, whereas Ply only inhibited the classical pathway [127]. In addition, LytA recruited both downregulators of the complement system (C4BP and FH) and cleaved the C3b and iC3b components bound to the pneumococcal surface demonstrating a critical role of LytA in evasion of complement-mediated immunity and phagocytosis [127].

The protective response of LytA, when inoculated in the lungs of a mice model, was seen in the 90s [35,125]. Mice vaccinated with LytA via intranasal immunization, produced both IgGs and IgAs in the serum, and sIgA in the local area of immunization [94]. These antibodies protected against an intranasal and intraperitoneal *S. pneumoniae* challenge and were tested against serotypes 23F, 19F, 6A, 14 and 6B [94]. Moreover, sIgAs might decrease the colonization state, necessary for the pneumococcus to cause pneumonia and sepsis, which makes LytA an ideal candidate to be combined with other recombinant proteins to reach an ideal protein vaccine candidate [94].

Two concerns arise with LytA as a vaccine candidate. The first is that the commensal *S. pseudopneumoniae* shares a LytA-like protein with the pneumococcus, making the bacteria a target by LytA antibodies, which could alter the balance of the community. The second is that sIgAs can affect the colonization state and, as discussed before, could prepare the niche for other pathogens.

#### 6.1.2. LytB

The *N*-acetylglucosaminidase LytB is a CWH with many roles in virulence. In spite of exhibiting noticeable polymorphism in the number of repeats encoding the choline-binding repeats, LytB is a well-conserved protein [115]. LytB is involved in nasopharynx colonization [96] and is the responsible of cell separation after the cell cycle, being localized at the sites near of the poles of the cell [128]. Moreover, it plays a role in the formation and structure of biofilms [95]. The role of LytB in evasion is more complex. This CBP does not directly affect the binding of C3b in the bacterial surface, but it enhances the ability of LytC to avoid the opsonization by C3b. In terms of phagocytosis, LytB diverted the phagocytosis mediated by alveolar macrophages and human neutrophils [96].

The hydrolase LytB was first proposed as a vaccine candidate in 2001, where Wizemann and colleagues observed that it conferred protection to a lethal challenge in a mouse sepsis model [129]. In addition, a detailed characterization of LytB as a potential vaccine candidate was more recently demonstrated, confirming that vaccination of mice with LytB elicited a strong immunological response, with high levels of IgG of different subclasses including IgG1, IgG2a, IgG2b and IgG3. As a result, these antibodies increased the activation of the classical pathway through C1q inducing the opsonization of C3b on the bacterial surface of different serotypes. These antibodies had functional consequences, as they trigger the phagocytosis process by human neutrophils. Finally, vaccination of mice with LytB increased bacterial clearance and induced protection against pneumococcal sepsis and invasive pneumonia caused by pneumococcal strains of serotypes 3 and 23F [77]. From the pathogenesis perspective, antibodies to LytB block one of the major functions of LytB, which is cell-daughter separation, and incubation of pneumococcal strains with LytB antibodies resulted in long chain formation [77]. These results make LytB another ideal vaccine candidate that could be combined with other antigens in a pneumococcal vaccine, as combinations of pneumococcal proteins can provide additive and synergistic effects beneficial to the immunization.

There are no data of this vaccine candidate reducing the nasopharyngeal carriage, but as the protein has an active role in colonization, antibodies against LytB could alter in some way the carrier state by *S. pneumoniae*. Moreover, the studies mentioned are based on full-length proteins; however, the LytB catalytic module contains three structurally independent domains [85], and all three are necessary for its optimal adhesion activity to respiratory epithelial cells [130]. Possibly, the use of epitopes located in any of these domains could be incorporated into a vaccine based on multiple antigens.

#### 6.1.3. LytC

Another well-known CWH, LytC, is an autolytic lysozyme that cleaves the *N*-acetylmuramoyl-*N*-glucosaminyl residues of the polysaccharide chain of the bacterial cell wall [131]. The *lytC* gene was detected in 100% of nasopharyngeal samples and was also the gene with the highest level of expression (>10^4^ copies/ml) of the human nasopharynx [132]. Between the many functions of LytC, an impotant one is its role in colonization [96], biofilm formation [97] and its noteworthy participation in cellular fratricide [133]. LytC has the ability to avoid the binding of C3b in the bacterial surface and diverts the phagocytosis mediated by both alveolar macrophages and human neutrophils [96].

In a work using serum antibodies from convalescent patients and from parents of young children, LytC was mainly identified by serum pools obtained from parents of young children [80]. As none of these sera donors were colonized by *S. pneumoniae*, it was hypothesized that the antibodies found in this group were from mucosal exposure to the pneumococcus from their children and could be considered potentially protective against nasopharyngeal colonization [80]: the first step toward invasive disease. They did not continue to work with LytC for animal protection studies, as it was not preselected with the applied criteria [80]. Other works show that immunization of mice with LytC increased the survival in comparison to the placebo group, although the immunological mechanism responsible for this protective effect was not explored [129].

#### 6.1.4. PspA

The pneumococcal surface protein A, PspA, is one of the most studied CBPs, as this protein is present in all clinically relevant serotypes, although it shows variability among them [88,89]. PspA is divided into two major families that are classified based on the amino acid divergence of the helical region before the proline-rich region (the most variable region of PspA) denoting it as a clade-defining region [89]. PspA interferes with the host’s immune response by inhibiting complement deposition on the bacterial surface [134,135,136].

The main role of PspA is interfering with the deposition of C3b in pneumococci and inhibiting the formation of a fully functional alternative pathway C3 convertase, thus altering the function of the alternative pathway, affecting the effective clearance of *S. pneumoniae* [134]. Moreover, it was also shown that it inhibits C-reactive protein and prevents the deposition of C4b, limiting complement deposition via the classical pathway [137].

Due to its variability, PspA as a vaccine candidate can only work if it has at least one PspA of each of the two major families, as less cross-reactivity between families than within families has been observed [138].

In a mice model challenge to *S. pneumoniae*, PspA elicited a protective response in the absence of other pneumococcal antigens [103]. Moreover, it showed to elicit protection against otitis media in a rat model [139], and immunization with a native full-length PspA virtually eliminated nasal carriage [140]. This made PspA an ideal vaccine candidate despite the problem of the variability within the protein family. Furthermore, PspA was found to be highly immunogenic in a study using serum from convalescent patients [80], where most patients developed antibodies against this protein.

A first clinical trial with a recombinant clade 2 PspA from the strain Rx1 was performed to evaluate not only safety but cross-reactivity with other pneumococcal strains with PspA of other clades [138]. They observed a reaction of anti-Rx1 sera against clade 1 antigens, given the similarity to clade two, but a reaction also to clades 3, 4 and 5 which was unexpected. There was an increase in reactivity of post immune sera to clade 6 antigen when the highest dosage of vaccine was administered, but this increase was minor. The same reactions of the post-vaccination sera were observed using whole-cell strains instead of the antigens with the different PspA clades [138]. Despite being safe and immunogenic, low sequence homology of PspA with human cardiac myosin raised concerns about PspA eliciting autoantibodies that could promote cardiac inflammation or autoimmune disease [141].

A PspA vaccine candidate was also approached using *Salmonella enterica* Typhi (*S. typhi*) vaccine vectors producing PspA. Despite being safe and well-tolerated in a phase I clinical trial, immunogenicity was limited possible due to pre-existing cross-reactive antibodies to *S. typhi* [142].

Both initial vaccines did not pass to the next phases of clinical trials, but there is a new phase Ia clinical trial to preliminarily evaluate the safety and immunogenicity of a protein-based pneumococcal vaccine that combines different PspA and Ply antigens (Clinical Trial Number, NCT04087460).

After the problems encountered with PspA, such as its low sequence homology with human cardiac myosin that could produce autoantibodies, the variability between strains, the *S. typhi* vectors not being immunogenic, etc., a full-length PspA as a protein vaccine candidate does not seem useful. Although, epitopes of the protein could be used in a combined protein vaccine.

#### 6.1.5. PspC

The major adhesin of pneumococcus is PspC, (CbpA, SpsA, PbcA), which has a high variability at the sequence level and allelic variations classifying this protein within 11 different groups [87]. Although its organization contains the same domains, each one has a unique sequence [90], and this antigenic variation within the PspC protein has been shown to affect immune evasion [143]. This protein is an important virulence factor that intervenes in different aspects of the pathogenesis process.

PspC is involved in adhesion and colonization of the nasopharynx and in the binding to the polymeric immunoglobulin receptor [104,144]. In terms of host immune evasion, PspC interacts with the complement system at different levels, being involved in the recruitment and binding to the complement regulatory FH [145,146,147,148] and even the downregulator of the classical pathway C4BP, although in this case in a PspC allele-dependent manner [98]. In addition, this protein prevents lysis mediated by the terminal complement complex (TCC) through its binding to vitronectin, inhibiting the deposition of the TCC [149,150].

In a mouse model, immunized with a PspC fragment, it was observed that anti-PspC antibodies inhibited FH binding to the bacterial surface, activated the complement classical pathway and improved opsonophagocytosis [106]. Another study showed that with nasal immunization, pneumococcal nasopharyngeal colonization decreased [151]. Besides, PspC has shown protection in animal model against IPD employing protective peptide epitopes from it [152]. In previous studies, the PspC peptide–L460D pneumolysoid fusion protein was tested using passive and active immunizations conferring protection against pneumococcal infection. In addition, the fusion protein was more broadly immunogenic than pneumolysoid alone, and antibodies were active in vitro against pneumococcus, *N. meningitidis* and *Haemophylus influenzae* [153].

It is interesting that a study that carried out passive immunization with hyper-immune sera containing specific polyclonal antibodies (anti-PspA serum, anti-PspC serum, anti-ClpP serum) showed that the protection obtained was greater in mice treated with the three sera than that obtained with the individual sera, observing a synergistic effect [154]. This makes PspA and PspC two CBPs that could be combined in a pneumococcal protein vaccine, using immunogenic peptide epitopes from both proteins.

However, there are studies using mouse models of infection with disparate results when evaluating the host’s immune response using this protein against pneumococcus; in some cases PspC has shown results of low efficacy or even not protection at all [155,156].

In convalescent humans, it has been observed that anti-PspC IgG antibodies block the pneumococcal adherence when the levels of FH are low, but when these levels are high, pneumococcal adherence is greater because there are more interactions between PspC and FH [157]. Anti-PspC antibodies acquired in natural immunity do not recognize the FH binding sites of PspC, possibly because the FH coats the bacteria during colonization, covering PspC and producing an inefficient antigen capture and presentation of these regions of PspC [157] resulting in non-acquisition of specific antibodies. Feasibly, immunization with a vaccine containing these epitopes would help the recognition of PspC at the time of colonization by interfering with the FH coating and avoiding immune evasion.

The FH binding motif is located within the hypervariable domain in the N-terminal region [158]. For this reason, there are differences between the strains that express the different variants; some are more efficient in FH binding and complement inhibition on the bacterial surface than others [159]. As previously discussed in PspA, this great variety of structures presented in this family of proteins makes it difficult to design a vaccine that protects efficiently against all the strains. It may happen that selective pressure selects the variants not included in the vaccine, making difficult to include this protein in the design of a new vaccine as it could present a low efficacy.

### 6.2. Other CBPs as Vaccine Candidates

#### 6.2.1. PcpA

PcpA is a promising vaccine candidate among the CBPs that has attracted attention in recent years [160,161,162,163,164]. PcpA, also known as CbpN, is involved in adherence to the host surfaces as it is part of LRRTP subfamily of proteins with leucine-rich repeats at the N-terminal module [107,108]. PcpA is found in virtually all clinical isolates studied so far, playing and important role in the establishment of pneumococcal pneumonia [107,165].

This protein has been proposed as a vaccine antigen because immunization of mice with PcpA elicits protection against pneumonia and sepsis, although it did not affect nasopharyngeal colonization [108]. Additional options of cocktail antigens including PcpA has been studied including PhtD and PlyD1 [163]. This trivalent pneumococcal protein vaccine (PPrV) conferred protection in infant mice against lethal pneumonia challenges using serotypes 3 and 6A; this protective effect being associated with an increased neutrophil-mediated phagocytosis in the lungs [161,164] and also protection in an AOM model [166].

Higher IgG and IgA antibodies against PcpA, as well as the protein antigens PhtD and Ply that compose the PPrV, were associated with a reduced AOM caused by the pneumococcus in children [167]. Moreover, antibodies against PcpA were found in patient convalescent sera [80]. The above mentioned made PcpA a promising vaccine candidate.

The use of PcpA in combination with other proteins such as the pneumococcal histidine triad protein D to see additive or synergistic effects has started the phase 1 vaccine clinical trial demonstrating that both antigens are immunogenic and safe [168]. The trivalent PPrV vaccine candidate went forward to phase 1 clinical studies, where they saw that the vaccine was safe and immunogenic in toddlers, infants and adults, with an increase in the immunogenicity in infants when aluminum as an adjuvant was used [169].

As this protein is found in virtually all clinical isolates and antibodies against PcpA do not affect the colonization state, this is one of the most promising vaccine candidates as it would ensure protection for pneumonia and sepsis against a wide range of pneumococcal isolates without affecting the nasopharynx microbiota. Thus, PcpA contrast with other CBPs that could possibly target the pneumococcal carrier state.

#### 6.2.2. CbpD, CbpG and CbpM

CbpD is a murein hydrolase, with a role in *S. pneumoniae* fratricide and division, which is expressed during competence [109]; CbpD also assist in fratricide together with LytA, LytC and CibAB [110]. CbpG participates in colonization on the mucosal surface of the nasopharynx and sepsis [99]. CbpG has a dual role depending on if it is secreted, cleaving the host extracellular matrix or attached to the bacterial surface with a role in bacterial adherence. Finally, CbpM, a less studied choline-binding protein with limited data about it, was supposed to interact with the C-reactive protein of the complement [107], but was later demonstrated to have a binding property to fibronectin, involved in streptococcal attachment to epithelial cells [170]. Antibodies against CbpD were found in the sera of convalescent patients [80].

There is not a protein vaccine candidate using CbpD, but there is an approach of using immunity against *S. pneumoniae* close relative commensals such as *S. mitis* and finding antisera that cross-react against pneumococcus. Shekhar and colleagues observed that antisera from rabbits that received the *S. mitis* strain reacted against different serotypes of *S. pneumoniae*, later observing CbpD and CbpM as some of the proteins that cross-react with *S. mitis* antisera [171]. This phenomenon is considered normal as both share 80% of the genome and an 80% similarity between some of their CBPs such as CbpD. This approach of studying how immunity against commensals could protect against *S. pneumoniae* colonization and infection is interesting and gives us new protein vaccine candidates such as CbpD and CbpM that have not been explored before, and it would be interesting to develop other protein combination vaccine candidates [171]. Vaccine proteins that could target not only *S. pneumoniae* but also other commensals should be avoided, as commensal microbiota serve to check the growth and spread of organisms that have pathogenic potential [119].

The immunogenicity of CbpG was tested in a mouse model, where all mice developed anti-CbpG antibodies that protected against colonization and systemic infection [172]. Later, a recombinant protein CbpG vaccine candidate was again proposed as it elicits high protection in mice challenged with a serotype 19F strain. Anti-CbpG antibodies increased neutrophil mediated opsonophagocytosis, the main route of pneumococcus clearance, which makes CbpG a suitable vaccine candidate [112], and interesting due to its dual role in mucosal and bloodstream compartments [99]. In the same study as CbpG, CbpM showed to elicit protection in mice challenged with serotype 19F strains, making it a suitable vaccine candidate, and both CbpG and CbpM could be combined for a future protein vaccine candidate [112].

## 7. Conclusions

The advances in the knowledge of the mechanisms of action of these proteins, together with the molecular DNA techniques, allow us to select highly conserved antigenic regions within the virulence proteins and combine different antigenic epitopes from more than one protein in a vaccine based on multiple antigens. Additionally, the antibodies generated against these antigens could block the mechanism of action of the proteins included in the vaccine.

As each of these proteins participates in certain pathogenic processes, we must take into account if the vaccine seeks to prevent pneumonia and IPD or also interfere with colonization, and this will depend on the relevance of the protein in question in each of these processes. The suitability of eliminating the carrier status in human populations is under debate, due to the possible alteration that could occur in the microbiota by leaving free space in an ecological niche that could be occupied by other pathogenic microorganisms [119]. Given that the colonization of the nasopharynx in adults is 5–10%, the impact of a vaccine that eliminates the carrier status of this population could not seem relevant compared to the benefits of preventing pneumococcal-related diseases. On the other hand, it is known that the PCVs used in childhood vaccination do eliminate the carrier state and that they have helped to eliminate pneumococcal disease in the general population—a clear benefit of diminishing the carrier state. The available data on the replacement of pneumococcus by other pathogenic bacteria in the nasopharynx of the child population are scarce and need to be more extensively studied.

The knowledge of many of these CBPs such as CbpD, CbpG and CbpM is low and their implication in the pathogenesis needs to be studied in more depth, as well as the immunogenicity that they could present, before being included as a vaccine. Moreover, the variability presented in some of these proteins, such as PspA and PspC, or the results obtained in preclinical studies (PspC) or in clinical trials (PspA), means that they could be discarded as vaccine candidates. With existing preliminary data, a CBP that could be a good candidate is LytB, but it requires further studies. PcpA is the CBP with more promising results in both preclinical studies and clinical trials, which when combined with PlyD1 and PhtD in the PPrV makes a good protein vaccine.

To conclude, obtaining a universal vaccine that protects against pneumococcal disease without affecting the commensal microbiota would be ideal. This goal could be achieved by using epitopes derived from different pneumococcal proteins in a chimeric construct. Meanwhile, an intermediate step would be a vaccine based on the polysaccharides of the most prevalent strains conjugated to a pneumococcal protein that reinforces the individual immune response. In this review, we have focused the attention on CBPs as vaccine candidates because they are an important group of proteins involved in the pathogenesis process with relevant findings confirming their immunogenicity and protection against pneumococcal infection. The contribution of these proteins to virulence and the fact that antibodies elicited against many of them may block or interfere with their role in the infection process deserve further attention and to be considered within the arsenal of antigen candidates for a universal protein vaccine against *S. pneumoniae*.

## Figures and Tables

**Table 1:** Pneumococcal choline-binding proteins.

CBP	Role in Pathogenesis	Evidence as a Vaccine Agent
LytA	Cellular lysis, immune evasion and capsule shedding [91,92,93]	Yes [94]
LytB	Biofilm formation [95], colonization [96] and immune evasion	Yes [77]
LytC	Biofilm formation [97], colonization [96] and immune evasion	Yes [98]
Pce	Colonization, adhesion to epithelial cells and recruitment of host proteases [99,100]	No
PspA	Immune evasion [101] and binding to lactoferrin [102]	Yes [103]
PspC	Colonization [104], invasion [105] and immune evasion [98]	Yes [106]
PcpA	Adhesion and aggregation [107]	Yes [108]
CbpD	Plays a role in competence and fratricide [109,110]	No
CbpF	Regulator of the function of LytC [111]	No
CbpI	Adhesion and immune evasion [107]	No
CbpG	Proteolysis and adhesion [99]	Yes [112]
CbpM	Adhesion and immune evasion [107]	Yes [112]
CbpL	Invasion and immune evasion [107,113]	No
CbpK	Unknown function	No
CbpJ	Adhesion and immune evasion [113,114]	No

## Data Availability

The data presented in this study are collected from the cited literature.
